# Factors influencing road traffic accidents causing maxillofacial injuries in Nalgonda District: prospective survey of 366 cases

**DOI:** 10.5249/jivr.v15i1.1789

**Published:** 2023-01

**Authors:** Batchu Pavan Kumar, Vuyyuru Vidya Devi, Tapas Kumar Bandyopadhyay, Syed Mehmood Hussaini

**Affiliations:** ^*a*^ Department of Oral and Maxillofacial Surgery, Kamineni Institute of Dental Sciences, Nalgonda, India.

**Keywords:** Road Traffic Accidents, Trauma, Injury, Alcohol

## Abstract

**Background:**

Road traffic accidents are the leading etiological factor for maxillofacial trauma in India. The incidence of these accidents is impacted by various cultural, socioeconomic, and behavioral factors the understanding of which is paramount in assessing their importance in influencing the incidence of maxillofacial injuries.

**Methods:**

Data was collected via a questionnaire from 366 patients who reported with maxillofacial injuries due to Road Traffic Accidents to the casualty and maxillofacial OPD at a tertiary center in the Nalgonda District over a five-year period. Data collected included patient details, type of vehicle involved, speed of the vehicle, type of accident, location of the accident, the seating of the patient, presence of alcohol influence, usage of helmet or seatbelt and the injuries sustained by the patient. Statistical analysis was done using Chi squared test.

**Results:**

88.5%of the patients were male and 87.4% of the cases were injured in RTA involving two-wheeler vehicles. (50.3%) of the accidents took place between 6 pm to 12 am. 41.5% of cases reported their speed at the time of the accident as 40- 60 kmph. 42% of accidents were reported as skid accidents. 70.29% of accidents on rural roads occurred at night (between 6 pm and 6 am) as opposed to 29.71% during the day. Only 4.37% of cases reported wearing seatbelts or helmets. 51.17% of the participants who were driving reported or were observed as being under the influence of alcohol.

**Conclusions:**

The poor conditions of the roads, the lack of use of protective measures while driving, and the high incidence of driving under the influence of alcohol were seen to be the most significant contributing factors to road traffic accidents causing maxillofacial injuries in the Nalgonda population.

## Introduction

Maxillofacial trauma, resulting in both hard and soft tissue injuries, have a large social impact in terms of the physical, economic and psychological burden they create. When associated with cerebral trauma, they are a major cause of mortality and morbidity, especially in developing countries. The incidence and etiological causes for maxillofacial trauma vary vastly by geographical region due to various cultural, socioeconomic, behavioral and legislative factors. The leading etiological factor for maxillofacial trauma in India was reported as road traffic accidents followed by interpersonal violence and other factors such as falls, sports injuries, industrial accidents, etc.^1^

Road traffic accident (RTA) can be defined as “An event that occurs on a way or street open to public traffic; resulting in one or more persons being injured or killed, where at least one moving vehicle is involved”. It can either be a collision, occurring between vehicles, between a vehicle and a pedestrian, animals or structures, or loss of control of the vehicle due to some other reason. The incidence of RTA and the factors involved in it vary from region to region. RTAs have been cited by WHO as the 6th leading cause of death in India.^2^

The maxillofacial region is the second most commonly injured site in road traffic accidents after extremities accounting for around 19% of RTA injuries.^3^ RTA is the leading cause of maxillofacial injuries in developing countries followed by interpersonal violence. As evident in multiple studies across different countries and populations, various factors, both personal and social influence, both the incidence of RTA and the injuries caused by it. In this study, we have recorded the various factors involved in the Maxillofacial injuries caused by Road Traffic accidents in the regional population around the Nalgonda area.

## Methods 

Data was collected from 366 patients who reported with maxillofacial injuries due to Road Traffic Accidents to the casualty and maxillofacial OPD at a tertiary center in the Nalgonda District, over a five-year period. Patients reporting with other causes of injury, like accidental falls or assaults were not included in the study. Patients with other injuries and patients who were unstable and requiring immediate intervention were not included due to the inability to collect accurate information from such patients.

Data was collected based on the information reported by the patient or their attenders according to the questionnaire, along with the clinical examination of the attending surgeon. Data collected included patient details, type of vehicle involved, speed of the vehicle, type of accident, location of the accident, the seating of the patient, presence of alcohol influence, usage of helmet or seatbelt and the injuries sustained by the patient.

Data was analyzed using Chi-squared tests for correlation of various factors in SPSS software Version 26.

## Results

A total of 366 patients, who reported to the casualty and OMFS OPD with injuries due to road traffic accidents, were included in the study out of which 324 (88.5%) were men and 42 were women (11.5%). The mean age of the participants was 32.67±13.89 years with a majority of them (37.3%) belonging to the 18-25 year-age group followed by 22.2% from the 25-35 year-age group. Greater than 65 years were the least involved age group at 2.7%

297 (81.14%) of 366 cases were reported to be driving at the time of the accident, of which 284 (95.62%) were men and 13 (4.38%) were women. The mean age of men who were driving was 31.92±12.10 years and of women who were driving was 22.84±3.64 years. Of the remaining cases, 66 (18.03%) cases were noted to be passengers, including 34 men (56.06%) and 29 women (43.94%). The remaining 3 cases were pedestrians. Of the 66 passengers, 16 were travelling in four-wheelers, 10 in three-wheel vehicles and 40 on two-wheeler vehicles. 

**Time, Speed and Location**

185 of the accidents (50.3%) took place between 6 pm to 12 am, 85 accidents (23.2%) occurred between 12 pm and 6 pm, 56 accidents (15.3 %) between 6 am to 12 pm and 40 accidents (10.9 %) between 12 am and 6 am, accounting for a total of 61.4% accidents at night.

152 cases (41.5%) reported their speed at the time of the accident as 40- 60 kmph, 144 cases (39.3%) as 60-80 kmph and 58 cases (15.8%) as 80-100 kmph. Only 8 cases (2.2%) were reported to be driving at greater than 100 kmph. One case was involved in a collision at less than 40 kmph. Speed was indeterminate in the case of the 3 pedestrians, as the driver was not available, but based on the patient descriptions, they were all categorized as less than 40 kmph. 73.33% four-wheelers were driving at speeds greater than 60kmph and 85.62% of two-wheelers were driving between 40-80 kmph.

92 cases, accounting for 42.2% of skid accidents reportedly occurred at 60-80 kmph, whereas 67 cases accounting for (50.37%) of the collision cases, were reported to have occurred at 40-60 kmph. Of the 210 cases that were reported as driving at speeds greater than 60 kmph, 72 cases (34%) occurred between 12 pm to 6 pm and 6 pm to 12 am each, with 16% and 15% occurring between 12 am to 6 am and 6 am to 12 pm, respectively. 

The cases showed almost equal distribution of the location of the accident with 110 cases (27.3%) occurring on the National Highway, 128 (35%) cases occurring on the State Highway and 138 (37.7%) occurring on rural roads. 70.29% of the accidents on rural roads occurred at night (between 6 pm and 6 am) as opposed to 29.71% during the day. Both National and State Highway showed a similar distribution of day and night accidents at 44% and 56% , and 43.75% and 56.25% respectively. 43% of all nighttime accidents occurred on rural roads. (Table 1 ) The correlation between the type of road and the time of the accident was seen to be statistically significant. (p=0.026).

52% of the cases that took place on the National Highway were travelling at speeds of 60-80 kmph, 41.7% of the cases on the State Highway and 51.79% of cases on rural roads took place at speeds of 40-60 kmph.

**Table 1:** Accidents on different types of roads at each time period.

		Time				Total
		12am- 6 am	6 am -12 pm	12 pm - 6 pm	6 pm - 12 am	
Road	National Highway	16	20	24	40	100
	State Highway	2	24	32	70	128
	Non- hihgway	22	12	29	75	138
Total		40	56	85	185	366

**Vehicles, Protection and Type of RTA**

30 patients (8.2%) sustained injuries in RTA involving car collisions. Out of these, 11 patients were driving, 16 were passengers and 3 were pedestrians hit by a car. 16 of the patients (4.4%) were injured in accidents involving Auto-rickshaws, including 6 drivers and 10 passengers. 320 patients (87.4%) were injured in RTA involving two-wheeler vehicles including both with and without gear motorcycles. Of these 280 patients were driving and 40 were pillion riders. 

Of the 366 cases, 134 cases (36.6%) were involved in vehicular collisions (including 3 cases of collisions involving pedestrians), and 232 cases (63.4%) were involved in accidents due to skidding of the vehicle. Among them, of the 30 patients involved in four-wheeler injuries 26 cases (86.67%) were involved in collisions with other vehicles and 4 (13.34%) were involved in collisions with roadside barricades/dividers that were categorized as skid for the purpose of the study. All 16 cases in the three-wheeler group were involved in collisions with other vehicles. In the two-wheeler group, 228 cases (71.25%) were involved in skid-type RTA and 91 cases (28.44 %) cases were involved in collisions. The type of RTA showed similar distribution across all three types of roads and the co-relation was not statistically significant.

Only 16 of the 366 patients (4.37%) reported following safety rules. 2 patients driving four-wheeler vehicles reported wearing seat belts and 14 patients driving bikes were wearing helmets all of whom were male. None of the passengers was following any safety measures of wearing helmets or seatbelts. 

**Alcohol**

Driving under influence of alcohol was recorded on the basis of self-reporting by the patient or the attender or by the doctor if the patient was still under influence at the time of reporting to the hospital. Of the 297 cases who were driving, 152 (51.17 %) cases reported or were observed as being under the influence of alcohol. The 18-25 years old age group were the highest represented among cases of driving under influence of alcohol at 37.5% followed by the 36-45-year age group at 28.94%. Overall 18-45 years age group accounted for 88.1% of cases under influence of alcohol (Table 2 ). 

**Table 2:** Cases of driving under influence of alcohol per age group.

		Age Group							Total
		0-17 years	18-25 years	26- 35 years	36-45 years	46-55 years	56-65 years	>65 years	
Alcohol	Yes	0	56	26	44	6	6	6	144
	No	20	76	62	26	22	12	4	222
Total		20	132	88	70	28	18	10	366

The incidence of driving under the influence of alcohol was highest at 75% in the cases that reported RTA between 12 am to 6 am followed by 45.4% in cases between 6 pm and 12 am (Table 3 ). 

**Table 3:** Cases of driving under influence of alcohol at each time period.

		Time				Total
		12am- 6 am	6 am -12 pm	12 pm - 6 pm	6 pm - 12 am	
Alcohol	Yes	27	3	35	79	144
	No	13	53	50	106	222
Total		40	56	85	185	366

Of the 152 cases of driving under the influence of alcohol, 127 (83.55%) cases involved skid accidents and 25 (16.44%) cases involved collisions. 63 (41.44%) of them reported driving at a speed of 60-80 kmph, 49 (32.22%) at 40-60 kmph and 36 (23.68%) at 80-100 kmph.

**Type of Injuries**

140 (38.3%) of the 366 cases presented only with soft tissue injuries while 226 cases (61.7%) presented with a combination of soft tissue and hard tissue injuries. 

Among soft tissue injuries, 38 cases (10.4%) showed only abrasions, 50 cases (13.7%) showed only contusions 172 cases (47%) showed lacerations and the rest 29% showed a combination of abrasion and lacerations. 

Hard tissue injuries were present in 184 (61.34%) of the 300 cases who were driving and in 42 (63.63%) of the 66 passengers. Among the hard tissue injuries recorded ZMC fractures were the most common at 27.4% followed by individual and combination mandible fractures (Symphysis/Parasymphysis/Body) at 25.7% (Table 4).

**Table 4:** Distribution of fractures by anatomical region among hard tissue injuries.

Fractures		
	Frequency	Percent
Dentoalveolar fracture	16	7.1
Mandible Symphysis/ Parasymphysis/ Body fracture	28	12.4
Mandible Condyle fracture	10	4.4
Combination (more than one) mandible fractures	30	13.3
Zygomatic arch fracture	18	8.0
ZMC fracture	62	27.4
NOE/Nasal fracture	26	11.5
Frontal fracture	4	1.8
Lefort fractures (alone and in combination with other fracture)	26	11.5
Panfacial Fractrures	6	2.7
Total	226	100.0

## Discussion

Road Traffic Accidents are the leading cause of maxillofacial injuries in India.^4^ Boffano et al. in their review of 30 years of publications note that almost all Asian studies have placed RTA as the most common of etiologies for maxillofacial injuries at 45-50%.^5^ As per data from the report on Road Accidents in India, in 2018, the number of accidents per 100,000 population, although reduced in the past few years, was still high at 36 in 2018. According to the Road traffic accident report by the Ministry of Road Transport and Highways, India, Transport Research Wing, 19,172 accidents were reported in Telangana in 2020.^6^ The incidence of RTAs, the maxillofacial injuries caused, and the regional variation seen in their patterns are influenced by various factors that a complex and often codependent. 

Gender distribution has shown an overwhelming predisposition towards male patients to a ratio of 7.14:1 which is marginally higher than some other studies reported.^2,7,8^ A strong male predisposition has been noted in other similar studies which is attributed to social factors leading to males being more active participants in public life and thus being more involved in traffic accidents both as drivers and as riders.^2,9^ Furthermore only 13 of the women involved were driving all of whom fell into an age group of less than 31 years. Yet even among the younger population the distinct predisposition in genders involved in RTAs remained persistent and was seen to be greater than seen in similar studies from urban populations.^10^

The average age of the patients in our study was 32.23 ±13.97 years with around 80% falling between the ages of 18 to 45 years old. The leaning of the mean age towards the younger population is reported across other studies and while part of it can be attributed to the youth heavy nature of the general population it also an indication that a greater number of the younger population having access to vehicles and a greater tendency toward more reckless driving and substance abuse. ^6,11^

The majority of the accidents took place after 6 pm and before 12 am. There was also a distinct increase in the number of accidents that took place after nightfall on rural roads compared to the day-night difference on National and State Highways. This could be attributed to regional factors of poor road maintenance and paucity of proper street lighting in rural areas. During daytime and in overall numbers, the accidents showed almost equal distribution among the 3 types of roads.

The majority of the cases in our study self-reported driving at the speeds of 40-60 kmph followed by 60-80 kmph. Both State Highways and rural roads showed a majority of the accidents to be caused at 40-60 kmph speed whereas on National Highways marginally more accidents took place at 60-80 kmph. There was a distinctly low number of high-speed accidents travelling at or above 100 kmph in the study. As the data of the study was derived from maxillofacial injury patients, it was considered that this was due to the fact that injuries involving high-speed accidents had a higher rate of mortality or multi-organ injury and thus, did not report to the Maxillofacial OPD/Casualty.

An overwhelming majority of the cases that presented with these maxillofacial injuries involved two-wheelers indicative of the regional socioeconomic conditions leading to greater use of two-wheelers as the preferred vehicle. However, this combined with the lack of enforced use of helmets in rural regions contributed to the majority of the injuries on the State Highways and rural roads. There was very low use of safety measures among both two wheeler and four-wheeler cases. It was noted that none of the women involved in two-wheeler accidents was wearing a helmet consistent with previous reports of low use of helmets among female accident victims.^6^

Most of the cases involved skid accidents rather than collisions reflective of poor road maintenance, reckless driving and the high level of substance abuse the cases included in the study. The low traffic flow in the region compared to urban regions also contributed to this, similar to findings in studies in other rural regions.^12^ Additionally, though the information was not included in the results as it was not a part of the study design, a substantial number of cases who reported skid accidents self-described them as being caused by an attempt to avoid passing obstructions on the road in the form of pedestrians and animals. About 20 patients reported animals including dogs, goats, cows and buffaloes, on the road as the cause of the accidents. Of the collisions, four bike collisions had occurred with animals including dogs and a pig. The disorganized habits of pedestrians in walking and crossing the roads and the presence of domesticated and non-domesticated animals on the road is a significant factor in RTAs in rural regions. 

Only 3 cases reported in our study were pedestrians and as such were not considered for separate analysis. However, it has been noted that pedestrian injury varies from vehicular injuries in the mechanism and type of injury and deserves further exploration.^13^

Alcohol was a high contributing factor in the accidents in the study with over half the participants admitting to having been driving under influence of alcohol. The lack of appropriate checks on driving under influence in rural areas is evident in the results. 18 to 25 and 35 to 45 years olds were found to be the most common group driving under influence of alcohol. Other studies have reported about a third of their population samples as under influence but did not make a clear distinction between drivers and passengers.^14^ Our study shows a much higher rate of driving under influence. The NFHS-5 Report (2019-2021) records alcohol consumption in rural areas in Telangana as 49%, much higher than the national average of 19% in rural areas.^15^ Cases that were reported after midnight showed the highest rate of driving under influence of alcohol followed by cases that were reported between 6 pm to 12 am. The post-midnight lack of scrutiny and late-night increase in consumption of alcohol are likely reflected in the results.

Laceration and combination of lacerations and abrasions were found to be the most common soft tissue injuries and ZMC fractures were seen to be the most common hard tissue injures. Specific co-relations of the etiological factors to the type of injury was found to be non-significant in the current sample and suggests scope for further exploration in the future. 

Other influencing factors were identified during the course of the study like the day of the accident (working day or weekend), traffic flow in the area of accident and distance of travel during the accident that were not included in the original assessment. As such the data collected on them was inconsistent and could not be analyzed but it must be noted that these aspects deserve further exploration in the future. Road traffic accidents are a major cause of maxillofacial trauma. As much as the prevention of road traffic accidents focuses on mortality, its role in maxillofacial trauma and resulting morbidity and its resulting financial and emotional cost is critical. A better understanding of factors affecting road traffic accidents focused on the specific regional realities is critical for establishing better public education programs and health care infrastructure. The study has further scope in correlating the individual factors to the demands on the management and its infrastructural abilities for future development.
